# *Pneumocystis jirovecii* Pneumonia in Kidney Transplant Recipients Receiving Belatacept: A Report of Two Cases With Atypical Presentations

**DOI:** 10.1016/j.xkme.2024.100891

**Published:** 2024-08-14

**Authors:** Elmar Pieterse, Jakko van Ingen, Wilbert van der Meijden

**Affiliations:** 1 Department of Nephrology, Radboudumc, Nijmegen, The Netherlands; 2 Department of Medical Microbiology, Radboudumc, Nijmegen, The Netherlands

**Keywords:** Kidney transplantation, *Pneumocystis jirovecii* pneumonia (PJP)

## Abstract

Immunosuppressive therapy after kidney transplantation is associated with an increased risk for the development of opportunistic infections, such as *Pneumocystisjirovecii* pneumonia (PJP). Belatacept, a selective costimulatory blocker that prevents T cell activation, was previously suggested to be a potential risk factor for PJP development in kidney transplant recipients. We present 2 cases of kidney transplant patients with PJP discovered unexpectedly during a diagnostic work-up for fever of unknown origin. Both patients lacked typical clinical findings such as hypoxia, ground-glass pattern on computed tomography, or suggestive biochemical alterations such as high lactate dehydrogenase levels or hypercalcemia. PJP should therefore be included in the differential diagnosis when evaluating fever in kidney transplant recipients receiving belatacept, even in the absence of typical pulmonary and laboratory findings.

## Introduction

*Pneumocystis jirovecii* pneumonia (PJP) is a potentially life-threatening infection in kidney transplant patients. Although prophylaxis has drastically reduced PJP incidence, it is still as high as 0.4%-2.2%.^1^ Well-known risk factors for the development of PJP include increased donor or recipient age, lymphopenia, previous cytomegalovirus infection, or prior treatment for graft rejection.^2^^,^^3^

PJP is typically considered when a hypoxic or coughing immunocompromised individual shows ground-glass pattern, reticular opacities, or septal thickening and pneumatoceles on high-resolution computed tomography (CT). Low lymphocyte counts, an elevated lactate dehydrogenase level, or hypercalcemia may be clues that further suggest PJP.^4^

Recently, we admitted 2 kidney transplant recipients for the analysis of fever of unknown origin with no pulmonary symptomology at presentation. Both were using belatacept. Belatacept was recently proposed to be a potential additional risk factor for PJP development in kidney transplant recipients.^5^

## Case 1

The first patient was a man in his 30s with a known history of IgA nephropathy requiring kidney transplantation from an unrelated living donor. His serum creatinine after kidney transplantation was 1.70 mg/dL (endogenous creatinine clearance 73 mL/min). He was cytomegalovirus seronegative. Approximately 4 years after kidney transplantation, his serum creatinine had increased to 2.83 mg/dL, with a kidney biopsy showing chronic antibody-mediated rejection as well as signs of an active component. De novo donor-specific antibodies were present in his serum. He was treated with plasmapheresis, alemtuzumab, and immunoglobulins and was given PJP prophylaxis for 6 months. Because kidney histology also showed signs of tacrolimus toxicity, he continued immunosuppression in the form of mycophenolate mofetil (MMF; 2 × 360 mg) and prednisone (7.5 mg), and 6 months later, belatacept was added due to a repeatedly low area under the curve of MMF and a slowly progressing decline in transplant function.

**Figure 1:**
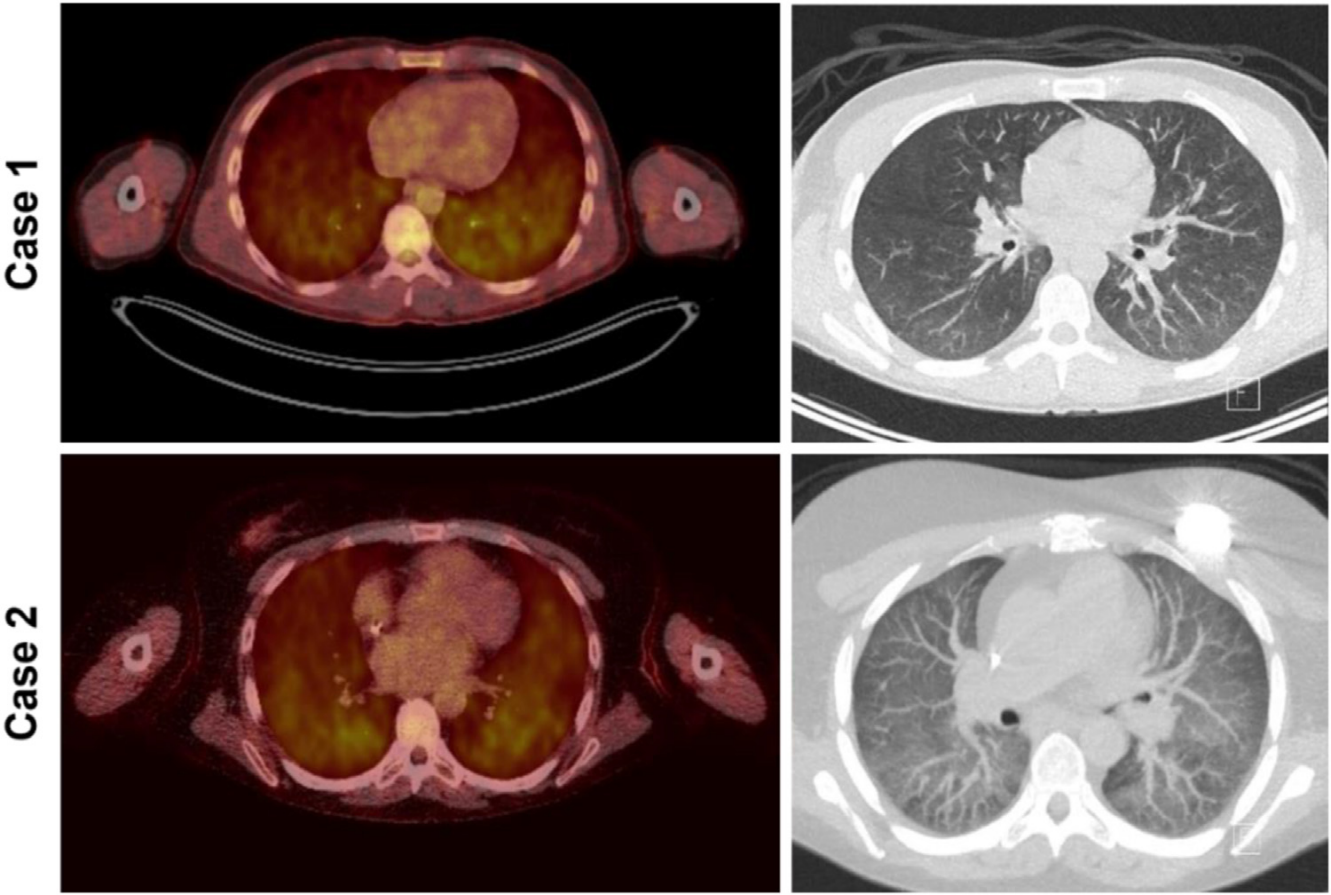
Representative PET-CT scan findings of case 1 (upper panels) and case 2 (lower panels). CT, computed tomography; PET, positron emission tomography.

**Figure 2:**
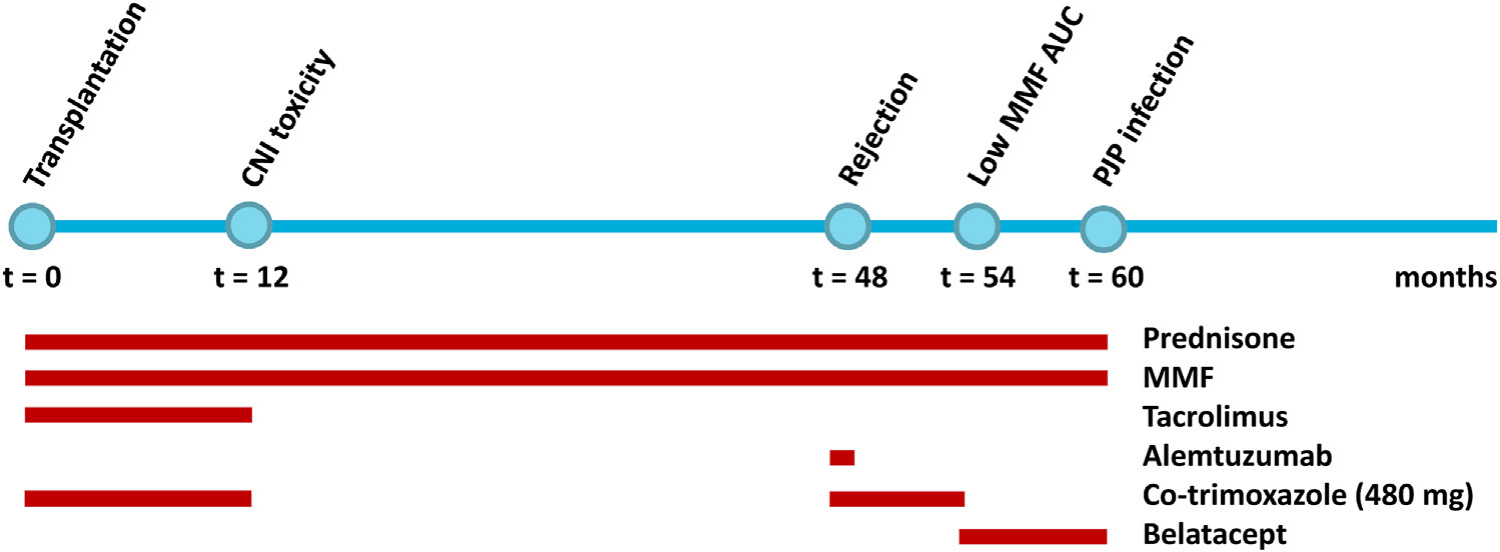
Time course of therapy modalities and PJP development in case 1. AUC, area under the curve; CNI, calcineurin inhibitor; MMF, mycophenolate mofetil; PJP, *Pneumocystis jirovecii* pneumonia.

A few months later, the patient was admitted to our inpatient clinic for the analysis of a fever with unknown cause. He had been struggling with fever for the past few weeks, earlier explained by “repeated viral infections” as a result of having a young child going to daycare. Blood and urine cultures were routinely negative. Biochemical analyses showed leukocytes 6.4 × 10^9^/L, lymphocytes 0.6 × 10^9^/L, baseline creatinine 3.34 mg/dL, lactate dehydrogenase 261 U/L, ionized calcium 1.35 mmol/L, and C-reactive protein 41 mg/L. Because of the absence of a clinical reason for his fever, a positron emission tomography (PET)-CT scan was performed, which showed diffuse increased fluorodeoxyglucose uptake across the lung parenchyma without consolidations, suggestive of medication-induced or viral pneumonitis (Fig 1). A bronchoalveolar lavage was performed, and microbiological analyses of a diluted low-volume sample only yielded a positive polymerase chain reaction result (PCR) for *P. jirovecii*, with a cycle threshold value of 32.28; a prior bronchial wash was PCR-positive with a cycle threshold value of 27,14. A serum β-D-glucan test was strongly positive (>600 pg/mL, cutoff >3.6), supporting the diagnosis of PJP. The patient was treated with a high dosage of trimethoprim/sulfamethoxazole, after which his fever disappeared. Due to repeated infectious episodes and progressive chronic transplant dysfunction, belatacept was discontinued, and dual therapy with MMF/prednisone was initiated (Fig 2). The patient is currently in screening for a new kidney transplant.

## Case 2

**Figure 3:**
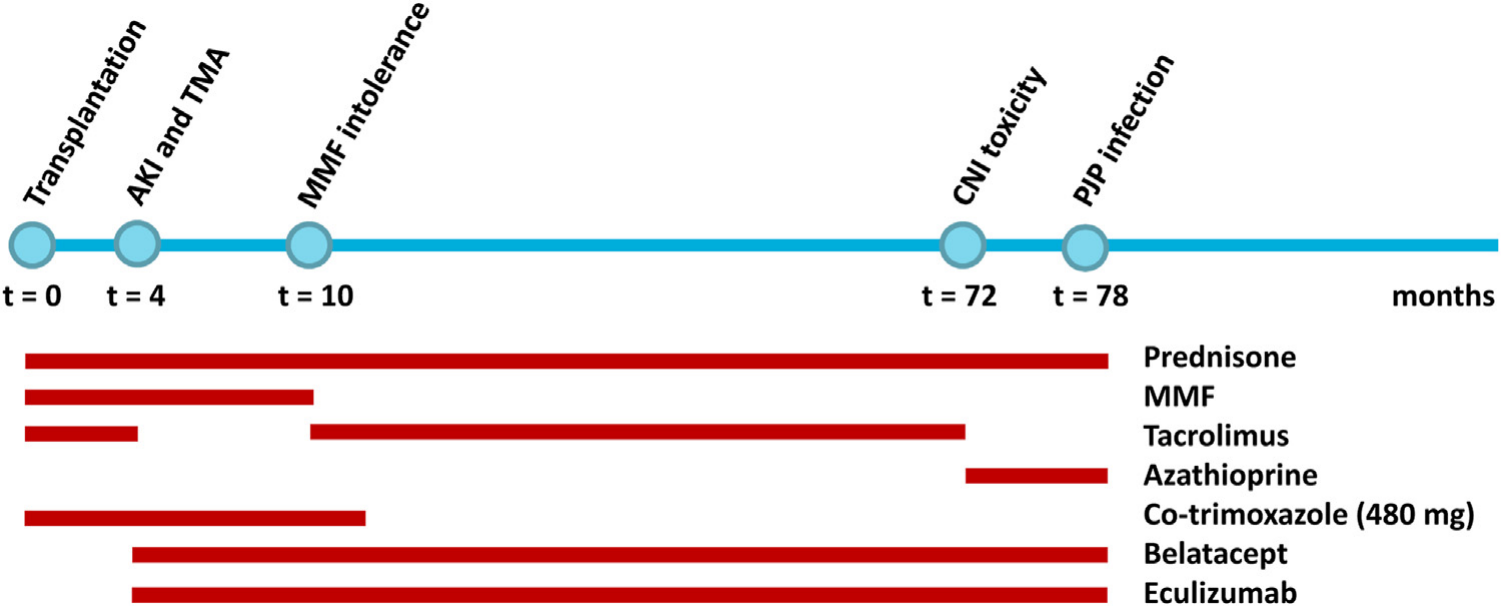
Time course of therapy modalities and PJP development in case 2. AKI, acute kidney injury; CNI, calcineurin inhibitor; MMF, mycophenolate mofetil; PJP, *Pneumocystis jirovecii* pneumonia.

The second patient was a woman in her late 40s with a known history of atypical hemolytic uremic syndrome due to a factor H mutation, requiring her second kidney transplant to be from an unrelated living donor. Her serum creatinine after kidney transplantation was 1.02 mg/dL (endogenous creatinine clearance 70 mL/min). She was cytomegalovirus seronegative. Approximately 6 years after kidney transplantation, she was admitted to our inpatient clinic because of ongoing fever for 1 week. She received eculizimab and—due to MMF intolerance and signs of tacrolimus toxicity in an earlier kidney biopsy—a triple immunosuppressive regime consisting of prednisone (5 mg), azathioprine (150 mg), and belatacept. Two months before admission, she had coronavirus disease 2019 with a relatively mild course, and she had been treated multiple times for urinary tract infections in the past few months, but blood and urine cultures were now negative. Biochemical analyses showed leukocytes 7.6 × 10^9^/L, lymphocytes 0.09 × 10^9^/L, baseline creatinine 1.98 mg/dL, lactate dehydrogenase 476 U/L, ionized calcium 1.28 mmol/L, and C-reactive protein 56 mg/L. Epstein Barr virus serology had recently shown mild IgM positivity, prompting a PET-CT scan to assess whether posttransplant lymphoproliferative disease was present (Fig 1). No signs of the disease were detected, but diffuse fluorodeoxyglucose avidity was observed in the lung parenchyma, suggesting medication-induced or viral pneumonitis. A bronchial wash tested positive for *P. jirovecii* by PCR with a cycle threshold value of 31.08; a subsequent bronchoalveolar lavage was performed 2 days later and was PCR-negative for Epstein Barr virus but again positive for *P. jirovecii* with a cycle threshold value of 39.65. The serum β-D-glucan test was also positive (74.4 pg/mL, cutoff >3.6), consistent with the diagnosis of PJP. The patient was treated with a high dosage of trimethoprim/sulfamethoxazole, and her clinical condition quickly improved. Belatacept was continued after discharge from the clinic and to date, no new infectious episodes have occurred (Fig 3).

## Discussion

Belatacept is a fusion protein produced in hamster cells by recombinant DNA technology that acts as a selective costimulation blocker of T cells, thereby inhibiting their activation. Standard immunosuppression for kidney transplant patients in our center includes induction with basiliximab followed by maintenance therapy with MMF, steroids, and calcineurin inhibitors. In cases of calcineurin inhibitor toxicity or intolerability to mTOR inhibitors or MMF, we increasingly consider belatacept.

Among the patients taking belatacept (n = 38), 5 (13%) had PJP in the last 5 years, including the 2 atypical cases we present here. Two of the 5 patients had previously been given anti-T cell therapy due to rejection, and all 5 were taking a triple immunosuppressive regimen. While a direct causal link between belatacept use and PJP cannot be made from this observation, it remains a striking finding. The first case of (fatal) PJP under belatacept was described in 2009,^6^ followed by 3 other PJP infections under belatacept in ABO-incompatible transplant patients reported in 2016 by Brakemeier et al.^5^ More recently, a large cohort study of late convertors to belatacept showed that 34 of 280 patients developed opportunistic infections, of which 28.6% were PJP.^7^ Whether belatacept use on its own is a risk factor for PJP development remains to be investigated. It is also possible that confounding factors such as age, prior anti-rejection therapy, or impaired transplant function, rather than belatacept itself, may explain this association.

Apart from the abovementioned epidemiological suggestions, the 2 cases we describe here are particularly worth sharing due to their atypical presentation with fever only, in which a pulmonary focus unexpectedly came to light on the basis of a PET-CT scan. In addition, the PET-CT scan showed diffuse fluorodeoxyglucose avidity in the lung parenchyma but lacked typical PJP findings. Instead, the radiologist suggested medication-induced or viral pneumonitis. Belatacept has been described to cause pneumonitis.^8^ It came as a surprise that in both patients, a bronchoalveolar lavage showed strong PJP positivity in PCR. It is unclear why patients taking belatacept actually fail to develop pulmonary symptomatology in response to PJP. CD4^+^ T cells are essential for clearance in PJP, as demonstrated by the high risk of human immunodeficiency virus-infected patients with a low CD4^+^ T cell count to develop PJP. It is theoretically plausible that an impaired CD4^+^ T cell-mediated proinflammatory adaptive immune response, because of its blockade by belatacept, may explain the lack of pulmonary symptoms. Taken together, we propose considering PJP in patients taking belatacept who present with fevers of unknown origin.
